# SNEV^P^ ^rp19/^ ^PSO^ ^4^ deficiency increases PUVA‐induced senescence in mouse skin

**DOI:** 10.1111/exd.12910

**Published:** 2016-03-10

**Authors:** Rossella Monteforte, Georg F. Beilhack, Reinhard Grausenburger, Benjamin Mayerhofer, Reginald Bittner, Regina Grillari‐Voglauer, Maria Sibilia, Hanna Dellago, Erwin Tschachler, Florian Gruber, Johannes Grillari

**Affiliations:** ^1^ Department of BiotechnologyUniversity of Natural Resources and Applied Life SciencesViennaAustria; ^2^ Christian Doppler Laboratory on Biotechnology of Skin AgingViennaAustria; ^3^ Division of Nephrology & DialysisInternal Medicine IIIMedical University of ViennaViennaAustria; ^4^ Neuromuscular Research DepartmentCenter of Anatomy & Cell BiologyMedical University of ViennaViennaAustria; ^5^ Evercyte GmbHViennaAustria; ^6^ Institute for Cancer ResearchMedical University of ViennaViennaAustria; ^7^ Department of DermatologyMedical University of ViennaViennaAustria

**Keywords:** DNA damage response, extracellular matrix, premature skin ageing, PUVA, senescence

## Abstract

Senescent cells accumulate during ageing in various tissues and contribute to organismal ageing. However, factors that are involved in the induction of senescence *in vivo* are still not well understood. SNEV^P^ ^rp19/^ ^PSO^ ^4^ is a multifaceted protein, known to be involved in DNA damage repair and senescence, albeit only *in vitro*. In this study, we used heterozygous SNEV ^+/−^ mice (SNEV‐knockout results in early embryonic lethality) and wild‐type littermate controls as a model to elucidate the role of SNEV^P^ ^rp19/^ ^PSO^ ^4^ in DNA damage repair and senescence *in vivo*. We performed PUVA treatment as model system for potently inducing cellular senescence, consisting of 8‐methoxypsoralen in combination with UVA on mouse skin to induce DNA damage and premature skin ageing. We show that SNEV^P^ ^rp19/^ ^PSO^ ^4^ expression decreases during organismal ageing, while p16, a marker of ageing *in vivo*, increases. In response to PUVA treatment, we observed in the skin of both SNEV^P^ ^rp19/^ ^PSO^ ^4^ and wild‐type mice an increase in *γ*‐H2AX levels, a DNA damage marker. In old SNEV^P^ ^rp19/^ ^PSO^ ^4^ mice, this increase is accompanied by reduced epidermis thickening and increase in p16 and collagenase levels. Thus, the DNA damage response occurring in the mouse skin upon PUVA treatment is dependent on SNEV^P^ ^rp19/^ ^PSO^ ^4^ expression and lower levels of SNEV^P^ ^rp19/^ ^PSO^ ^4^, as in old SNEV ^+/−^ mice, result in increase in cellular senescence and acceleration of premature skin ageing.

## Introduction

Cellular senescence is a stress‐inducible permanent growth arrest contributing to organismal ageing 1, 2, 3, 4. Senescent cells accumulate in various tissues with ageing and contribute to disrupt tissue structure and function because of factors released in their secretome referred to as ‘senescent‐associated secretory phenotype’ (SASP) 3, 5, 6, 7. Indeed, the accumulation of senescent cells *in vivo* is associated with age‐related pathologies in numerous tissues, while clearance of senescent cells in mouse tissues delays the onset of age‐associated disorders 8. How cellular senescence *in vivo* is induced and which factors might play a role in this process, however, are still not well understood, even if emerging evidence suggests that mitochondrial oxidative stress promotes cellular senescence *in vivo* 9.

One model for studying stress‐induced cellular senescence in the context of ageing is 8‐methoxypsoralen (8‐MOP) in combination with broad‐band ultraviolet A (320–400 nm) (PUVA) treatment on the skin, because a prominent side effect of repeated PUVA therapy is premature ageing of the skin 10, 11, one form of an acquired premature progeroid symptom (APPS) 11. PUVA treatment is widely and successfully used as treatment for skin diseases such as psoriasis and mycosis fungoides 12, where its therapeutic effect depends on the generation of DNA damage prevalently in form of interstrand cross‐links (ICL) 11, 13. Because ICL are formed by endogenously produced bifunctional agents like malondialdehyde (MDA), this model is certainly of interest, while it is clear that UV‐induced DNA damage and photoageing of the skin is clinically of high importance. PUVA treatment induces the formation of DNA damage foci at telomeres dependent on ataxia telangiectasia‐mutated and Rad3‐related protein (ATR) 14 and morphological and functional changes reminiscent of cellular senescence in human keratinocytes and fibroblasts 15, 16, 17, 18. The activation of ATR upon PUVA‐induced DNA interstrand cross‐link initiates and maintains the senescent phenotype in primary fibroblast 14.

The protein SNEV^Prp19/PSO4^ (for simplicity reason termed SNEV in the following) is one important factor at the intersection of interstrand cross‐link repair 19, the DNA damage response 20, 21 and cellular ageing 22, 23. In addition, SNEV is an essential pre‐mRNA splicing factor 24 and, moreover, involved in adipogenesis 25 and neurogenesis 26, 27. SNEV is induced upon DNA damage by treatment with ICL inducers cisplatin and mitomycin C, as well as by DNS single‐ and double‐strand break (SSB and DSB)‐causing reagents such as bleomycin or gamma irradiation 20. In addition, it interacts with several DNA‐modifying/repair factors including TdT 20, metnase 28, XAB2 29 and WRN, whereby WRN interaction is crucial during ICL repair 19. Among the most prominent interactors of the SNEV complex are the kinases ATR and ATM, the two master regulators of the DNA damage response. ATR directs the cellular response to damage inflicted by replication fork stalling agents such as UV, hydroxyurea and ICL inducers 30, and the SNEV complex is required for the activation of ATR‐downstream targets as well as to guarantee the establishment of the S‐phase checkpoint and cell survival upon UV and hydroxyurea treatment 19. In addition, SNEV is phosphorylated by ATM at serine 149 upon H_2_O_2_ treatment of Hela cells and fibroblasts. This phosphorylation is necessary to protect the cells from accumulating DNA breaks and from apoptosis upon oxidative stress‐ and cisplatin‐induced DNA damage and is in part responsible for delaying cellular senescence 23.

In terms of cellular senescence, SNEV ectopic overexpression in human endothelial cells extends the cellular lifespan by increasing resistance to oxidative stress 22. Conversely, decreased SNEV protein levels as observed in heterozygous mouse (SNEV^+/−^) embryonic fibroblasts (MEFs) accelerate entry into cellular senescence, while its full knockout results in early embryonic lethality 31. Although the role of SNEV in senescence and DNA damage repair has been described *in vitro,* little is known about its function *in vivo*. In this study, we used heterozygous SNEV^+/−^ mice as a model to elucidate the role of SNEV in DNA damage repair and senescence *in vivo*. For this purpose, we performed PUVA treatment on the skin of SNEV^+/−^ and wild‐type littermate control mice. We asked whether decreased levels of SNEV, as in heterozygous SNEV^+/−^ mice, result in increase in cellular senescence and accelerate premature ageing of the skin upon PUVA treatment.

## Materials and methods

### Animals

SNEV^+/−^ mice (generation of SNEV^+/−^ mice is described in Fortschegger et al. 31) and WT littermate controls (C57BL/6 background) were maintained with alternating 12‐h light/dark cycles at controlled temperature (22 ± 2°C) and humidity (50–55%) conditions. Water and food were provided *ad libitum*. Mice were age‐ and sex‐matched within each experiment. All animal procedures were approved by the Austrian Government.

### Genotyping by PCR

Mouse genotyping was performed according to the protocol described in Fortschegger et al. 31.

### PUVA treatment

SNEV^+/−^ and WT littermate control mice were divided in two groups according to the age: 3 months old (young) and 20 months old (old). The back of the mice was waxed 1 day before PUVA treatment. The mice were painted on their back with either 200 *μ*l of 8‐methoxypsoralen (8‐MOP) in ethanol (0.1 mg/ml) or 200 *μ*l of vehicle (95% ethanol). UVA irradiation was performed only on psoralen‐painted mice after 30 min using a Sellamed 3000 UVA‐1 therapy lamp (Sellas, Ennepetal, Germany) filtered for the emission at 340–400 nm at a distance of 20 cm from the dorsal skin of the mouse. The UVA dose used was 1.5 J/cm^2^. The treatment was performed three times a week for two weeks. The PUVA doses and the duration of the treatment were selected (as minimal dose inducing increase in DNA damage marker *γ*‐H2AX and increase in epidermis thickness) from preliminary experiments. At the end of the treatment (24 h after the last dose), a group of mice, consisting of half of the treated SNEV^+/−^ and half of the treated WT littermate control mice (age‐matched), was sacrificed (labelled as PUVA in the figures). The second half including treated SNEV^+/−^ and treated WT littermate control mice (age‐matched) was sacrificed 1 month after the treatment (labelled as 1 month after PUVA in the figures). Control group consisted of SNEV^+/−^ and WT littermate control, vehicle‐painted (age‐matched) mice (labelled as C in the figures). Each experimental group consisted of five mice. The experiment was repeated twice.

### Tissue collection

Dorsal skin (approximately 1 cm^2^) was excised and immediately fixed in 4% buffered formaldehyde for paraffin embedding. In addition, dorsal skin was frozen in liquid nitrogen for protein analysis.

### Histology

Paraffin sections of 5 *μ*m thickness were used for haematoxylin–eosin and Masson's trichrome staining. Haematoxylin–eosin (H&E)‐stained sections were randomly selected (from each animal, three sections were chosen) for the quantification of epidermal thickness. For each section, 15 visual fields were selected, and for each field, epidermis thickness (epidermis area) was measured using ImageJ program and normalized to epidermis length. The measurements were taken independently by two laboratory members. Masson's trichrome staining for collagen was performed using the Accustain Trichrome staining kit (Sigma‐Aldrich, St. Louis, Missouri, USA) according to the manufacturer's instruction.

### Immunohistochemistry

Paraffin sections of dorsal skin (5 *μ*m) were stained with Prp19/PSO4 (SNEV) antibody (IHC‐00120; Bethyl Lab, Montgomery, TX, USA). Immunohistochemistry was performed using Vectastain ABC kit (VectorLabs, Burlingame, CA, USA). Briefly, after deparaffinization, the sections were exposed to antigen retrieval in 1× citrate buffer pH 6.0 (Sigma‐Aldrich) according to the manufacturer's protocol. Endogenous peroxidase was blocked by incubating the sections in 0.3% H_2_O_2_ for 30 min at room temperature. The sections were incubated with diluted normal serum for 30 min at room temperature and then incubated with Prp19/PSO4 primary antibody (1:100) overnight at 4°C in a humidified chamber. Biotinylated secondary antibody was applied for 1 h at room temperature to the sections. ImmPACT DAB peroxidase substrate (VectorLabs) was used for antibody detection. After final dehydration, coverslips were mounted onto the slides using Permount mounting medium (Fisher, Vienna, Austria).

### Immunofluorescence

Paraffin sections of skin (5 *μ*m) were stained with phospho‐histone H2A.X (Ser139) (20E3) antibody raised in rabbit (Cell Signaling Technology, Danvers, MA, USA). After deparaffinization, the sections were exposed to antigen retrieval at sub‐boiling temperature for 10 min in EDTA buffer (1 mm EDTA, 0.05% Tween, pH 8.0). The sections were incubated with phospho‐histone H2A.X primary antibody (1:100) for 2 h at room temperature. Secondary goat anti‐rabbit antibody conjugated with Alexa Fluor^®^ 594 was applied 30 min at room temperature. Coverslips were mounted onto slides using Vectashield mounting medium with DAPI (1.5 *μ*g/ml) (Vector Laboratories).

The percentage of *γ*‐H2AX‐ and p16‐positive nuclei in the epidermal layer was calculated as described: five visual fields were selected for each section, and for each field, the total number of nuclei in the basal layer of the epidermis (DAPI‐stained blue nuclei) was counted. Then, the number of *γ*‐H2AX‐ and p16‐positive nuclei (red nuclei) was counted, and the percentage of *γ*‐H2AX‐ and p16‐positive nuclei/total number of nuclei in the epidermis was calculated. The measurements were taken independently by two laboratory members.

### Protein lysates and Western blot

Mouse skin was homogenized in ice cold lysis buffer (50 mm Tris–HCl, pH 7.5, 150 mm NaCl, 0.5% NP40, 20% glycerol) with freshly added proteases and phosphatases inhibitor cocktails (Roche, Vienna, Austria). The homogenate was centrifuged at 15 000 g for 30 min at 4°C. The supernatant was collected and the aliquots were stored at −70°C. The protein content was measured by Bradford assay. Equal amounts of protein were subjected to electrophoresis on 4–12% NuPage gels (Invitrogen, Carlsbad, CA, USA) in 1× MOPS running buffer and then transferred to PVDF membrane. The PVDF membranes were subsequently blocked with 3% non‐fat milk in 1% Tween 20–PBS buffer for 1 h at room temperature. PVDF membranes were incubated with the following primary antibodies: Pso4/Prp19 (SNEV) (1:1000, A300‐102A, Bethyl Lab); *γ*‐H2AX (1:1000, ab26350; Abcam, Cambridge, UK); p16^INK4a^ (1:200, SC‐1661; Santa Cruz); MMP‐13 (1:500, ab75606; Abcam); beta‐actin (1:10 000, A5441; Sigma‐Aldrich); and gapdh (1:1000, sc‐25778; Santa Cruz) overnight at 4°C. Thereafter, incubation with secondary antibodies Alexa 680 – (1:10 000; Life Technologies, Carlsbad, CA, USA) or Infrared 800‐conjugated (1:10 000; Lincoln, Nebraska, USA) was performed for 1 h at room temperature. Detection was performed using an Odyssey Infrared scanner, and the optical density of the bands was measured using Image Studio software vs. 2.1 (Licor).

### Statistical analysis

Data were presented as mean ± SD and were analysed using the Student's *t*‐test (unpaired, two‐tailed). *P* < 0.05 was considered statistically significant.

## Results

### SNEV expression decreases during chronological ageing of the skin

**Figure 1:**
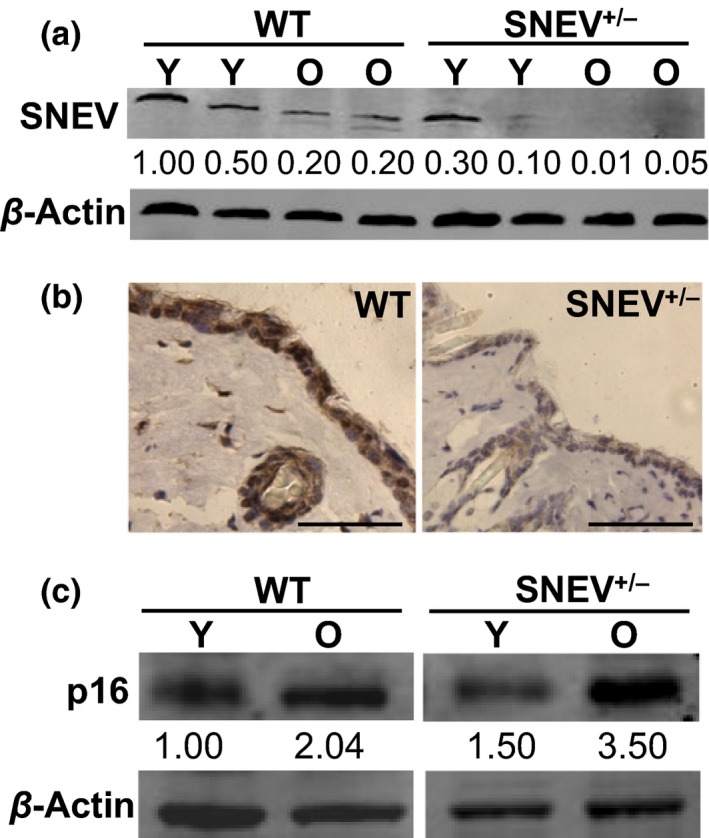
SNEV protein levels decrease during chronological ageing of the mouse skin. (a) Representative Western blot analysis for SNEV in young (Y) and old (O) WT and SNEV ^+/−^ mice. *β*‐Actin used as loading control. As shown in Fig. 1a, the levels of SNEV within young WT mice (1.00 and 0.50 O.D.) and SNEV ^+/−^ mice (0.30 and 0.10 O.D.) were variable; however, SNEV ^+/−^ mice showed SNEV levels three‐ to five‐fold lower as compared to WT mice; (b) representative photomicrographs of immunohistochemistry for SNEV on skin sections from young WT (left panel) and young SNEV ^+/−^ (right panel) untreated mice (scale bar: 50 *μ*m); (c) representative Western blot analysis for p16 in young (Y) and old (O) WT and SNEV ^+/−^ mice. *β*‐Actin as loading control. O.D., optical density.

Because SNEV levels decrease in various cell types during replicative senescence *in vitro* 22, we measured the levels of SNEV protein in the skin of both young and old WT and SNEV^+/−^ mice by Western blot in order to see whether it changes upon organismal ageing. Indeed, similar to cellular ageing, SNEV protein expression decreased upon organismal ageing. The levels of SNEV in the skin of old WT mice were 4.0‐fold lower as compared to young WT mice. Similarly, SNEV expression decreased in old SNEV^+/−^ mice to barely detectable levels by Western blot (Fig. 1a). As shown in Fig. 1a, the levels of SNEV within young WT mice and young SNEV^+/−^ mice were variable; however, SNEV^+/−^ mice showed 3.0‐ to 5.0‐fold lower levels of SNEV as compared to WT mice. SNEV localized to the nuclei of epidermal and hair follicle keratinocytes (Fig. 1b). Because it is known that senescent cells accumulate during chronological ageing of the skin 32, we measured the levels of p16, as a marker of cellular senescence *in vivo* 33, in the skin of both young and old WT and SNEV^+/−^ mice by Western blot. In the skin of WT as well as of SNEV^+/−^ mice, p16 levels increased by around 2.0‐fold and 2.3‐fold, respectively, during ageing (Fig. 1c). In old SNEV^+/−^ mice, p16 levels were higher (1.7‐fold) than in old WT mice. Thus, during chronological ageing of the mouse skin, SNEV expression decreases, while p16 levels increase.

### Photoprotective response to PUVA treatment is dependent on SNEV levels

**Figure 2:**
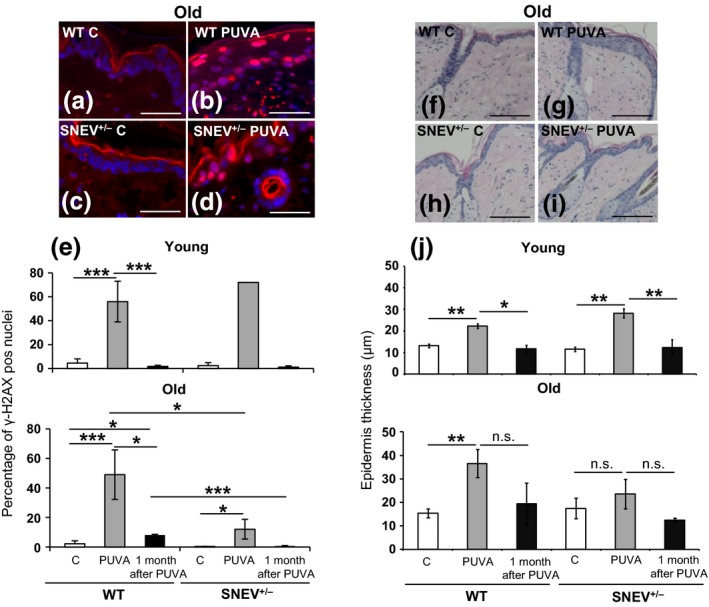
DNA damage response to PUVA treatment and analysis of epidermis thickness on H&E‐stained mouse skin sections. (a–d) Representative photomicrographs of *γ*‐H2AX immunofluorescence of old WT and SNEV ^+/−^ mouse skin (scale bar: 50 *μ*m); (e) percentage of *γ*‐H2AX‐positive nuclei in the epidermis as measured in the *γ*‐H2AX‐stained sections. Data shown are from one representative experiment, *n* = 3 mice per treatment group. ***P < 0.005; *P < 0.05; (f–i) representative photomicrographs of H&E‐stained paraffin sections of old WT and SNEV ^+/−^ mouse skin (scale bar: 200 *μ*m); (l) epidermis thickness (mean ± SD), as measured in the H&E‐stained skin sections. Epidermis thickness was measured using ImageJ program and normalized to the epidermis length. Data shown are from one representative experiment, *n* = 4 mice per treatment group. **P < 0.01; *P < 0.05; n.s., not significant; C, control.

In order to study the involvement of SNEV in DNA damage response and senescence *in vivo*, we performed PUVA treatment on young and old WT and SNEV^+/−^ mice as an inducer of DNA damage and premature skin ageing 10. We evaluated the DNA damage response to PUVA treatment by measuring the levels of *γ*‐H2AX, as a marker of DNA damage, known to accumulate in the cells after PUVA treatment 14. In young WT as well as young SNEV^+/−^ mice, the percentage of *γ*‐H2AX‐positive nuclei in the epidermis increased after PUVA treatment (up to 60% *γ*‐H2AX‐positive nuclei) as compared to untreated WT and SNEV^+/−^ controls (5% *γ*‐H2AX‐positive nuclei) (Fig. 2e). One month after PUVA treatment, the percentage of *γ*‐H2AX‐positive nuclei in the epidermis of young WT and young SNEV^+/−^ mice was down to baseline again (Fig. 2e). Old WT mice reacted as well to PUVA treatment with an increase in *γ*‐H2AX‐positive nuclei in the epidermis (up to 50% *γ*‐H2AX‐positive nuclei) as compared to the untreated control (Fig. 2a, b, e). One month after treatment, the percentage of *γ*‐H2AX‐positive nuclei in old WT mice was, however, still significantly higher (*P* < 0.05) as compared to the untreated old WT control (8% vs 2%) (Fig. 2e).

Surprisingly, in old SNEV^+/−^ mice, the increase in *γ*‐H2AX‐positive nuclei in the epidermis after PUVA treatment was blunted as compared to old WT mice (Fig. 2e), although still significantly higher (*P* < 0.05) (12% *γ*‐H2AX‐positive nuclei) as compared to the untreated old SNEV^+/−^ controls (1% *γ*‐H2AX‐positive nuclei) (Fig. 2c, d, e). One month after treatment, the percentage of *γ*‐H2AX‐positive nuclei in old SNEV^+/−^ mice was back to control levels (Fig. 2e). This might suggest an impaired DNA damage sensing/defensive capacity dependent on SNEV and ageing as has been observed in human PBMCs with age 34.

Furthermore, we measured the epidermal thickening in response to PUVA treatment. Epidermal thickening occurs upon photodamage and photoageing by UV radiation and may act as a defense mechanism 35, 36. PUVA treatment significantly increased (*P* < 0.01) the epidermis thickness of both young WT and young SNEV^+/−^ mice (22.30 ± 1.09 and 28.20 ± 2.10 *μ*m, respectively) as compared to young WT and young SNEV^+/−^ untreated controls (13.11 ± 0.85 and 11.52 ± 0.90 *μ*m, respectively) (Fig. 2j). The epidermis thickness returned to basal levels 1 month after PUVA treatment in young WT (11.78 ± 1.67 *μ*m) as well as in young SNEV^+/−^ (12.33 ± 3.60 *μ*m) mice (Fig. 2j). In old WT mice, the epidermis thickness significantly (*P* < 0.01) increased after PUVA treatment (36.61 ± 5.93 *μ*m) as compared to untreated WT control (15.40 ± 1.89 *μ*m) (Fig. 2f, g, j). Remarkably, in old SNEV^+/−^ mice, no significant increase in the epidermis thickness was observed after PUVA treatment (23.56 ± 6.36 *μ*m) as compared to untreated SNEV^+/−^ control (17.40 ± 4.35 *μ*m) (Fig. 2h, i, j), suggesting a reduced defensive mechanism associated with lower levels of DNA damage response. A reduced defensive response to PUVA treatment was observed exclusively in old SNEV^+/−^ mice where the levels of SNEV were considerably lower than in young SNEV^+/−^ mice. This indicates a potential threshold level of SNEV that would be needed for an appropriate defensive response.

### PUVA‐induced increase in p16 and MMP‐13 as well as ECM degradation depends on SNEV levels

**Figure 3:**
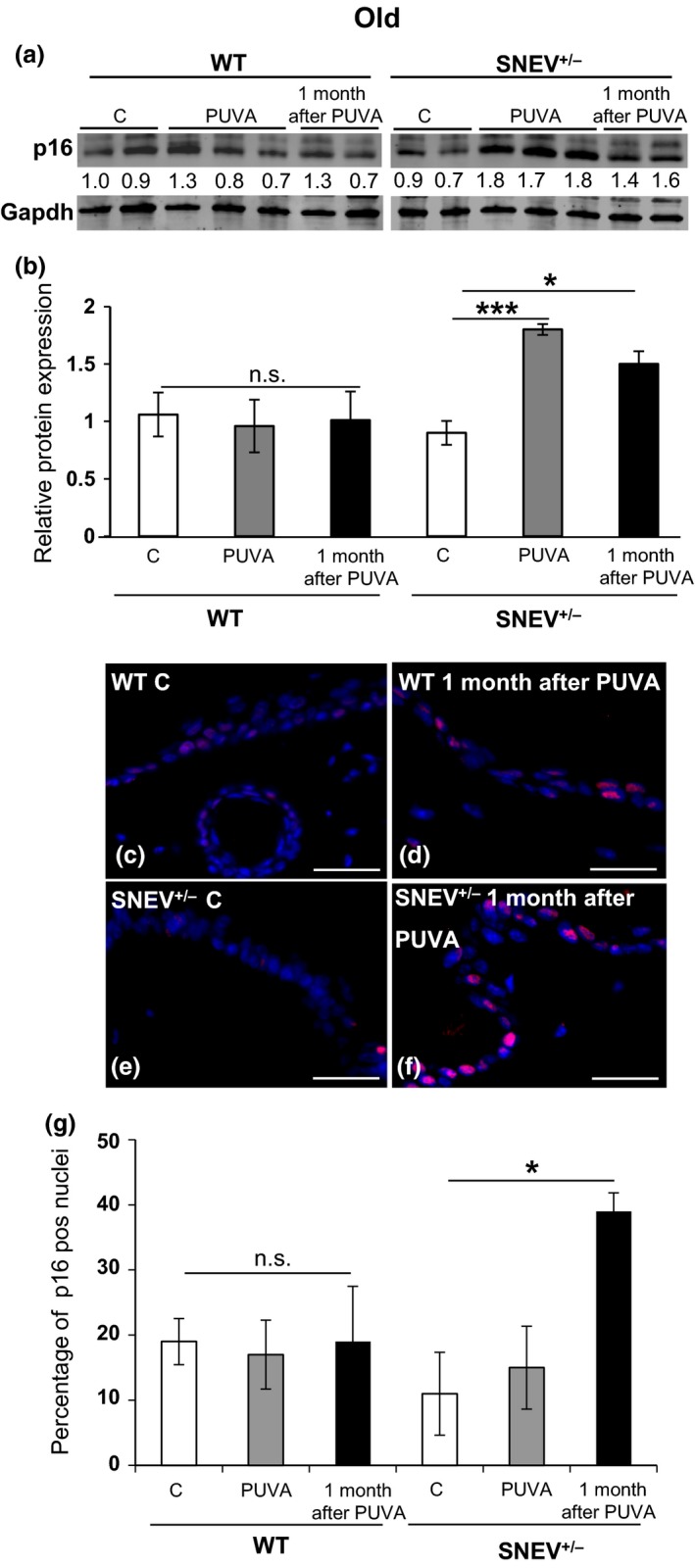
Levels of p16 increase in the skin of old SNEV ^+/−^ mice upon PUVA treatment. Representative Western blot analysis of p16 in the skin lysates of old WT and SNEV ^+/−^ mice (a, b). Gapdh used as loading control. p16 and gapdh were scanned simultaneously. Bar graph (mean ± SD) is the average fold change of p16 protein normalized to gapdh (*n* = 3). ***P < 0.005, *P < 0.05; n.s. = not significant; (c–f) representative photomicrographs of p16 immunofluorescence of old WT and SNEV ^+/−^ mouse skin (scale bar: 50 *μ*m); (g) percentage of p16‐positive nuclei in the epidermis as measured in the p16‐stained sections. Data shown are from one representative experiment, *n* = 3 mice per treatment group. *P < 0.05; n.s., not significant; C, control.

**Figure 4:**
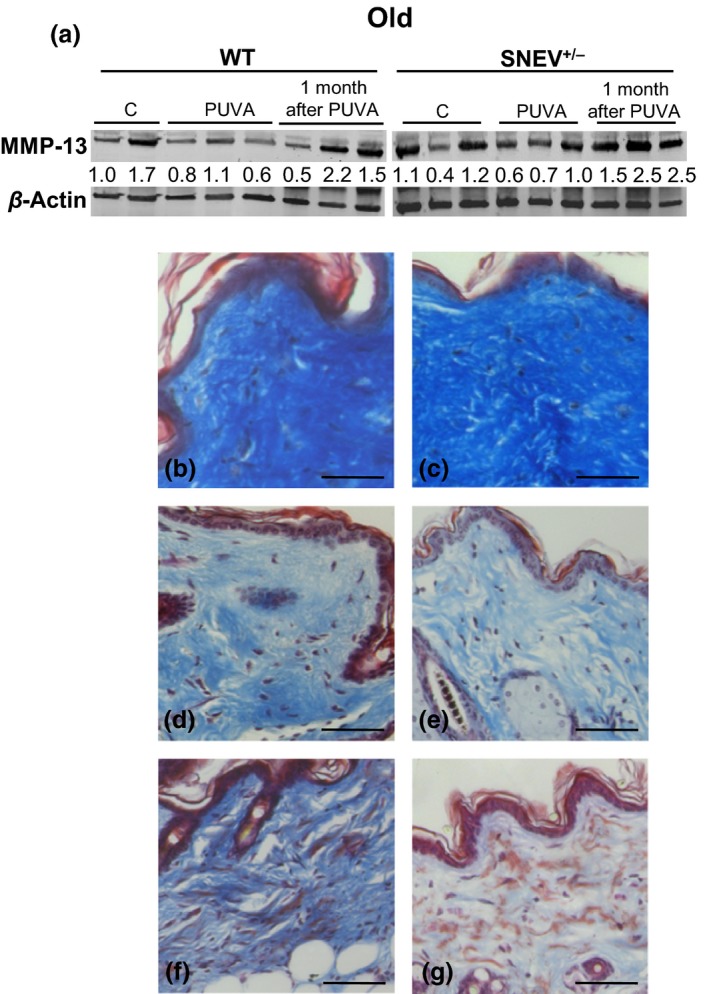
Collagen degradation occurs in the skin of old SNEV ^+/−^ mice upon PUVA treatment. (a) Representative Western blot analysis of MMP‐13 in old WT and SNEV ^+/−^ mice. *β*‐Actin used as loading control. MMP‐13 and *β*‐actin were scanned simultaneously; C = control; (b–g) representative Masson's trichrome staining on WT and SNEV ^+/−^ mouse skin sections (scale bar: 100 *μ*m): (b) young WT control; (c) young SNEV ^+/−^ control; (d) old WT control; (e) old SNEV ^+/−^ control; (f) old WT 1 month after PUVA treatment; (g) old SNEV ^+/−^ 1 month after PUVA treatment. Old SNEV ^+/−^ mice show 1 month after PUVA treatment (5 g) loose collagen fibres (light blue fibres) and signs of elastosis (red fibres).

Because SNEV levels decrease with chronological age and lower SNEV levels associate with a reduced defensive response to PUVA treatment, we asked whether old SNEV^+/−^ mice show signs of senescence and premature ageing of the skin upon PUVA treatment. For this purpose, we measured the levels of p16, an accepted marker of cellular senescence *in vivo* 33, 37, by Western blot and immunofluorescence staining. Furthermore, we measured the levels of metalloproteinase‐13 (MMP‐13), known to be secreted by senescent cells 38, 39, 40 by Western blot. In WT mice (young and old) as well as in young SNEV^+/−^ mice, p16 levels showed no changes after PUVA treatment as compared to untreated controls (Figs 3a, b and S1). Interestingly, in old SNEV^+/−^ mice, the levels of p16 significantly increased (*P* < 0.005) immediately after PUVA treatment and remained at significantly higher levels (*P* < 0.05) one month after PUVA treatment as compared to untreated controls (Fig. 3a, b). Immunofluorescence staining for p16 confirmed the significantly higher number of p16‐positive nuclei (*P* < 0.05) in the epidermis of old SNEV^+/−^ mice one month after PUVA (35%) treatment as compared to untreated controls (14%) (Fig. 3e, f, g). No significant increase in the percentage of p16‐positive nuclei in the epidermis of old SNEV^+/−^ mice was observed immediately after PUVA treatment as compared to untreated control (14%) (Fig. 3g). This difference between the results from the Western blot and the immunofluorescence staining might be due to the fact that for the Western blot, we used whole‐skin lysates and for immunofluorescence we counted exclusively the percentage of p16‐positive nuclei in the epidermis. In addition, the levels of MMP‐13 in old SNEV^+/−^ mice significantly increased (*P* < 0.05) one month after PUVA treatment as compared to untreated controls (Figs 4a and S2). No significant changes in MMP‐13 levels were observed in old WT mice upon PUVA treatment (Figs 4a and S2).

These data suggest that in old SNEV^+/−^ mice, cells positive for some senescence markers and therefore senescent‐like cells accumulate upon PUVA treatment and might lead to the degradation of extracellular collagen. In order to test for changes in the extracellular matrix (which might be due to collagen degradation as well as reduced collagen synthesis), we stained for collagen 41, 42 using Masson's trichrome staining. While Masson's trichrome staining revealed a similar collagen structure and density in untreated young SNEV^+/−^ and young WT mice, old SNEV^+/−^ mice showed one month after PUVA treatment thinner and more loosely organized collagen fibres and signs of elastosis as compared to equally treated old WT mice (Fig. 4b–g). Taken together, these data indicate that old SNEV^+/−^ mice accumulate senescent‐like cells and show signs of premature skin ageing and collagen degradation in response to PUVA treatment. In contrast, old WT mice show higher DNA damage response accompanied by increased defensive mechanisms and repair capacity.

## Discussion

Here, we show that premature ageing of mouse skin is dependent on SNEV levels. Indeed, lower SNEV levels, as in old SNEV^+/−^ mice, associate with impaired defensive response to PUVA treatment leading to senescence and premature skin ageing. In this study, we used PUVA treatment, known to induce DNA damage and premature skin ageing 13, 14, as a model to investigate the involvement of SNEV and interstrand cross‐link‐causing agents in the induction of cellular senescence in mouse skin *in vivo*. The involvement of SNEV in DNA damage response *in vitro* has been widely described 19, 20, 21, 23, 28, 43, as summarized in the introduction. It has been shown that the SNEV core complex directly interacts with WRN helicase and is essential in early steps of ICL repair *in vitro* 21. It is of note that WRN helicase is mutated in the human autosomal recessive disease Werner syndrome (WS) and that this segmental progeroid syndrome manifests as skin atrophy, wrinkling and hair greying 44, suggesting that unrepaired DNA damage in skin cells by the lack of DNA repair factors including SNEV and WRN contributes to accelerated ageing of the skin.

Here, we observed that the induction of defense mechanisms and the induction of cellular senescence upon PUVA treatment depend on SNEV levels.

Induction of defense mechanisms is blunted in the SNEV^+/−^ mice as observed by low numbers of *γ*‐H2AX‐positive cells immediately after PUVA treatment. An age‐dependent decrease in the induction of *γ*‐H2AX has recently been reported in human PBMCs in response to DNA double‐strand breaks 34. It is, however, entirely unclear how such a decrease might depend on SNEV. One possibility is that SNEV haploinsufficiency leads to premature skin ageing per se like in the PBMCs from elderly. Another possibility is that the lack of SNEV limits the formation of *γ*‐H2AX foci by impeding DNA damage signalling or by direct biochemical interaction even though cells have accumulated DNA damage. However, this seems unlikely in view to the finding by Marechal et al. that SNEV knockdown results in more persistent *γ*‐H2AX foci. Another possibility is that SNEV might be required for PUVA‐induced, ATR‐dependent formation of DNA damage foci, because it was recently reported that SNEV is required for the formation of the ATRIP complex, as well as for ATR‐mediated DNA damage‐response downstream signalling 45.

Old SNEV^+/−^ mice showed also absence of the defensive epidermal thickening in response to PUVA treatment 36. We speculate that cells which are not able to repair and/or sense the DNA damage induced by PUVA treatment would also fail to respond to the damage by epidermal thickening. Because cell proliferation and differentiation are necessary for epidermal thickening 46, we suggest that increased levels of p16, an accepted marker of senescent cells *in vivo* 8, 47, might contribute to the reduced epidermal thickening in old SNEV^+/−^ mice together with SNEV's activity in mitosis 48. This increase in p16 levels is well in accordance with other studies showing p16 increase during skin ageing 33, 37. Interestingly, p16 levels in old SNEV^+/−^ mice were higher than in the old WT mice, suggesting that lower levels of SNEV might be associated with higher accumulation of senescent cells. This idea is supported by our previous findings that SNEV expression decreases upon replicative senescence *in vitro* 22. After PUVA treatment, p16 levels rapidly increased in old SNEV^+/−^ mice and it is known that exposure of human dermal fibroblast to PUVA treatment results in replicative senescence 16, 49. Our results therefore indicate that the accumulation of senescent or at least senescence‐like cells in the skin occurring in response to PUVA treatment in *vivo* is dependent on SNEV expression. This is further supported by our observation that the skin of old SNEV^+/−^ mice shows high levels of the collagenase MMP‐13 and signs of collagen degradation in response to PUVA treatment. Because the secretome of senescent cells includes matrix‐degrading enzymes, specifically collagenases 38, 39, 40, the accumulation of senescent cells might contribute to the loss of collagen structure in the skin causing the prematurely aged skin phenotype. Taken together, our data suggest that upon PUVA treatment of the mouse skin, the DNA damage response is dependent on SNEV expression and that low levels of SNEV, as in old heterozygous SNEV^+/−^ mice, result in the accumulation of senescent cells and accelerated premature ageing of the skin, indicating that SNEV might be a factor necessary to counteract stress‐induced senescence of skin cells *in vivo*.

## Author contributions

RM designed and performed experiments, analysed data and wrote the paper; GB analysed and interpreted data and gave conceptual advice; RG analysed *γ*‐H2AX staining and interpreted data; BM performed *γ*‐H2AX immunofluorescent staining; RB contributed essential reagents and tools; RG gave conceptual advice and interpreted data; MS contributed essential reagents and tools; HD participated in writing; ET contributed essential reagents and tools and gave conceptual advice; FG contributed essential reagents and tools, gave conceptual advice and critically revised the paper; JG supervised the project, interpreted data and wrote the paper.

## Conflict of interests

JG and RGV are co‐founders of Evercyte GmbH.

## Supporting information


**Figure S1.** Representative Western blot analysis of p16 in young WT and SNEV^+/−^ mice. Gapdh used as loading control. p16 and gapdh were scanned simultaneously. Bar graph (mean ± SD) is the average fold change‐values of p16 protein normalized to gapdh (*n* = 3). n.s. = not significant.
**Figure S2.** Representative Western blot analysis of MMP‐13 in old WT and SNEV^+/−^ mice. Bar graph (mean ± SD) is the average fold change‐values of MMP‐13 protein normalized to β‐actin (*n* = 3). *P < 0.05; n.s. = not significant.
