# Supplementary material for: SNEVP rp19/ PSO 4 deficiency increases PUVA‐induced senescence in mouse skin
Source: Exp Dermatol. 2016 Mar 10;25(3):212–7. doi: 10.1111/exd.12910 (PMC4832318; doi:10.1111/exd.12910)
Supplement: Supplementary file 1 — Figure S1. Representative Western blot analysis of p16 in young WT and SNEV+/− mice. Gapdh used as loading control. p16 and gapdh were scanned simultaneously. Bar graph (mean ± SD) is the average fold change‐values of p16 protein normalized to gapdh (n = 3). n.s. = not significant. Figure S2. Representative Western blot analysis of MMP‐13 in old WT and SNEV+/− mice. Bar graph (mean ± SD) is the average fold change‐values of MMP‐13 protein normalized to β‐actin (n = 3). *P < 0.05; n.s. = not significant. [file EXD-25-212-s001.ppt]

## Slide 1
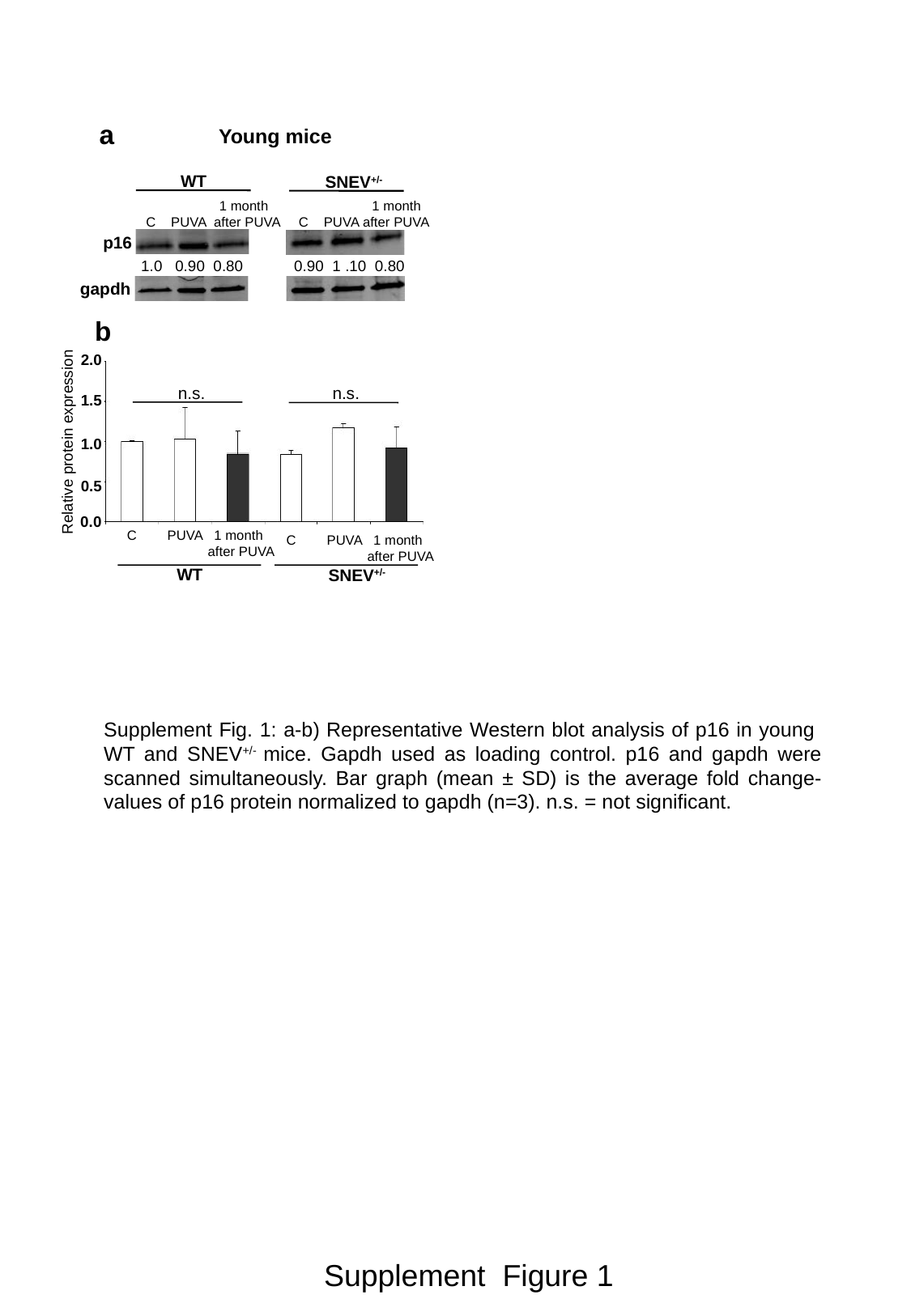

a
Young mice
WT
SNEV+/-
 1 month
 C PUVA after PUVA
 1 month
 C PUVA after PUVA
p16
 1.0 0.90 0.80 0.90 1 .10 0.80
gapdh
b
2.0
n.s.
n.s.
1.5
Relative protein expression
1.0
0.5
0.0
C PUVA 1 month
 after PUVA
C PUVA 1 month
 after PUVA
WT
SNEV+/-
Supplement Fig. 1: a-b) Representative Western blot analysis of p16 in young WT and SNEV+/- mice. Gapdh used as loading control. p16 and gapdh were scanned simultaneously. Bar graph (mean ± SD) is the average fold change-values of p16 protein normalized to gapdh (n=3). n.s. = not significant.
Supplement Figure 1

## Slide 2
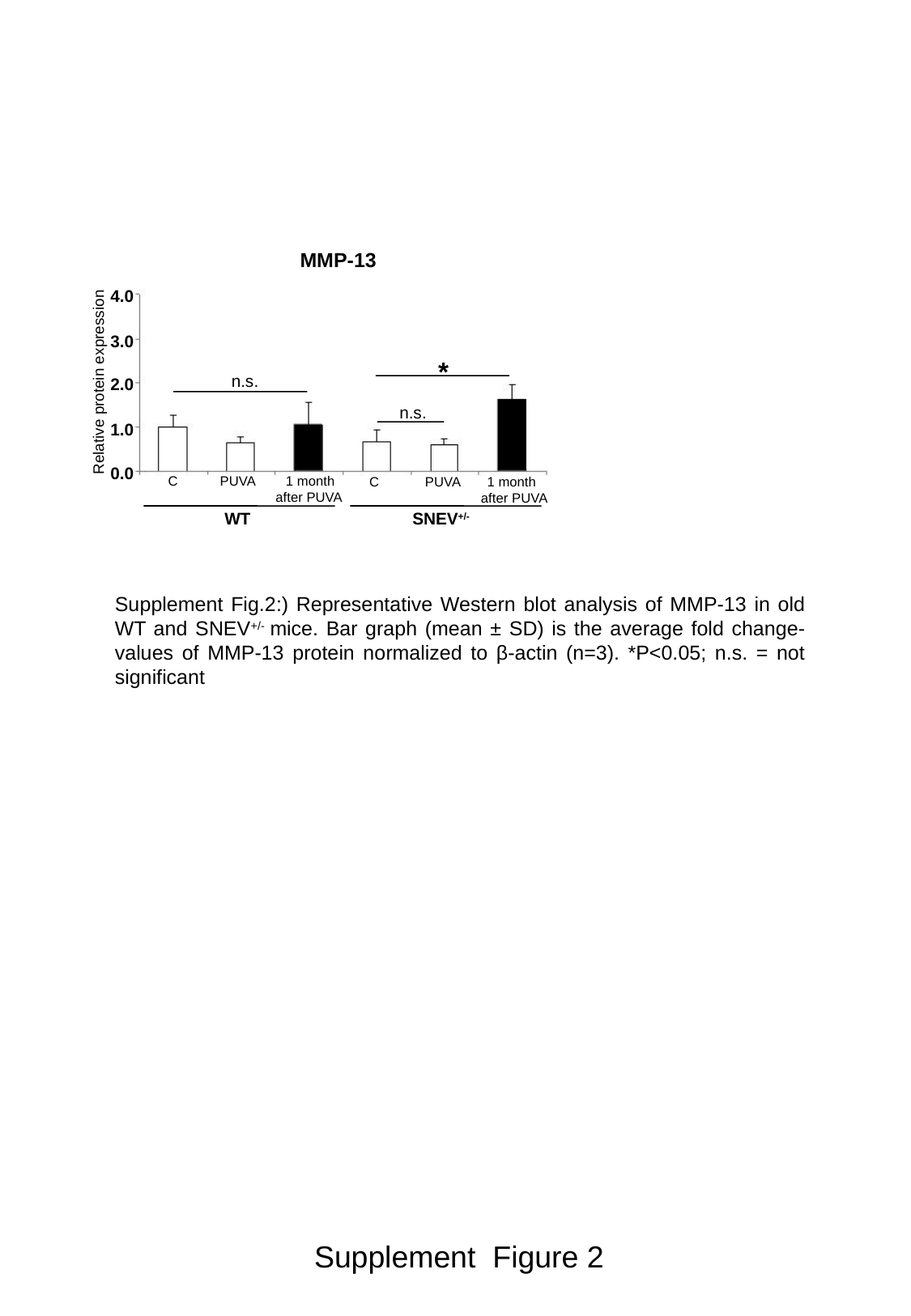

MMP-13
4.0
3.0
*
n.s.
Relative protein expression
2.0
n.s.
1.0
0.0
C PUVA 1 month
 after PUVA
C PUVA 1 month
 after PUVA
WT
SNEV+/-
Supplement Fig.2:) Representative Western blot analysis of MMP-13 in old WT and SNEV+/- mice. Bar graph (mean ± SD) is the average fold change-values of MMP-13 protein normalized to β-actin (n=3). *P<0.05; n.s. = not significant
Supplement Figure 2
